# Considerations, barriers and enablers of deprescribing among healthcare professionals in Ogun State, Southwest, Nigeria: a cross-sectional survey

**DOI:** 10.1186/s12913-024-11101-0

**Published:** 2024-05-24

**Authors:** Sule Ajibola Saka, Tolulope Ruth Osineye

**Affiliations:** Department of Clinical Pharmacy & Bio-Pharmacy, Olabisi Onabanjo University, Sagamu Campus, Nigeria

**Keywords:** Barriers, Enablers, Considerations, Deprescribing, Nigeria

## Abstract

**Background:**

Deprescribing is a clinical intervention aimed at managing polypharmacy and improving older adults’ health outcomes. However, evidence suggests that healthcare professionals (HCPs) may face challenges in implementing the intervention. This study aimed to explore the considerations, barriers and enablers of deprescribing among HCPs in Southwest Nigeria.

**Methods:**

A quantitative cross-sectional survey was carried out among consecutively sampled HCPs including physicians, pharmacists and nurses in two public tertiary healthcare hospitals in Ogun State, Southwest, Nigeria. A structured 43-item self-administered questionnaire was used to explore the participants’ sociodemographics, HCPs’ experience, considerations, barriers and enablers of deprescribing in older adults. The data were summarised using descriptive statistics including frequency and percentage. The Kruskal–Wallis test was used to determine differences in perceptions among the groups on a Likert scale. A *p*-value < 0.05 was considered significant.

**Results:**

Overall, 453 copies of the questionnaire were analysed. Of the participants 204 (45.0%) were within the age group of 20–30 years; 173 (38.2%) claimed that older adults occasionally requested deprescribing of their medications. The majority (417; 92.1%) considered patients’ quality of life to be very important in deprescribing; 423 (93.4%) opined that having a care goal known to members of the HCP team is an enabler for deprescribing while 308 (68.0%) disagreed or strongly disagreed that lack of incentives and remuneration for HCPs that de-prescribe is a barrier to deprescribing. There is a significant difference among the participants across professional groups on the assertion that pressure from pharmaceutical companies is a barrier to deprescribing in older adults (*p* = 0.037).

**Conclusions:**

The participants in this study had various considerations for deprescribing medication in older adults including patients’ quality of life. Having a care goal known to every HCP involved in managing a patient is an enabler for deprescribing while the lack of incentives and remuneration for HCPs that de-prescribe may not necessarily be a barrier to deprescribing. There is a need for regulations and policies to support the identified enablers among HCPs and reduce the barriers to effective deprescribing process.

**Supplementary Information:**

The online version contains supplementary material available at 10.1186/s12913-024-11101-0.

## Background

Deprescribing is defined as a systematic process of deliberate withdrawal or reduction in the dosage of inappropriate medications based on the currently available clinical evidence and within the context of an individual patient's preference and care goal [1, 2]. It is a process supervised by a healthcare professional to manage polypharmacy and improve patients’ health outcomes [3].

Polypharmacy, defined as regular use of at least five medication predisposes older persons to adverse drug reactions, increases care complexity and escalates the difficulty in therapeutic management apart from its negative impacts on older adults’ health outcomes [4, 5]. The negative health outcomes of polypharmacy in older persons may be further aggravated by the prescription of potentially inappropriate medications (PIMs) [6]. Globally, many multimorbid older patients are exposed to polypharmacy with PIMs in the course of seeking care in health facilities [7–9]. The high prevalence and negative impact of PIMs, coupled with the need to individualise medication therapy underscores the growing acceptance of deprescribing as a health intervention strategy in older patients [7, 8].

Despite the benefits of deprescribing however, studies have shown that the practice may face challenges from patients, healthcare systems and healthcare professionals. The willingness of patients to accept deprescribing is considered a great challenge. Patients may be apprehensive about their health conditions after deprescribing or about opposing their general practitioners’ prescriptions [10, 11]. Many barriers to deprescribing have been reported among HCPs including uncertainty concerning the effectiveness and safety of deprescribing, inadequate knowledge about the process, inadequate inter-professional collaboration, complexity of existing deprescribing guidelines, and the lack of remuneration for the HCPs that de-prescribe [7, 12–14]. The health system challenges include the clinical practice guidelines on the care of older persons with multimorbidity, diagnosis and prescribing culture, and decision-making in health care systems [15, 16].

On the other hand, share decision-making between patients and HCPs, the availability of tools or algorithms for deprescribing, acceptability of non-pharmacological alternatives by patients and improved collaborations among HCPs have been reported as enablers of deprescribing in clinical practice [16]. Although studies have been conducted on barriers and facilitators of deprescribing in developed countries, evidence suggests that this may not be translatable to resource-limited settings because most of the studies are likely to have recruited participants who are keen on practice transformation for deprescribing [17]. An understanding of the HCP’s perceptions of issues relating to deprescribing may assist in guiding further research on developing strategies and policies towards addressing the barriers and harnessing the enabling factors for the well-being of older adults.

In Nigeria, Pharmacists and Nurses are not legally allowed to prescribe medications but they are important in the success of any de-prescribing process. Deprescribing is a multidisciplinary intervention that involves different healthcare professionals, importantly the Pharmacists and Nurses [17, 18]. Pharmacists through medication review can identify medications that require deprescribing while Nurses can be a veritable asset in identifying especially in-patients that may benefit from the process and recommend to the prescribers. Many older adults in Nigeria are on polypharmacy with attendant negative health outcomes. While a few studies have evaluated the barriers to deprescribing among patients in Nigeria, [18, 19], very little is known about the considerations, barriers and enablers of deprescribing among healthcare professionals in the country. This study aimed to identify the considerations, barriers and enablers of deprescribing in older patients among healthcare professionals in Nigeria.

## Methods

### Study design and settings

A quantitative cross-sectional survey was carried out among consecutively sampled HCPs including physicians, pharmacists and nurses using a 43-item self-administered questionnaire. The study was carried out in the Consultant Outpatient Clinic, General Outpatient Clinic, Specialty Clinic, and Cardio-Renal Clinic of Olabisi Onabanjo University Teaching Hospital (OOUTH), Sagamu and Federal Medical Centre, (FMC), Idi-Aba, Abeokuta, Ogun State, Southwest, Nigeria. Olabisi Onabanjo University Teaching Hospital and FMC are among the public tertiary healthcare institutions approved for undergraduate and postgraduate residency training for physicians, as well as clinical training for other HCPs including pharmacists, nurses, and medical laboratory scientists in Nigeria. Presently, there is no hospital at the primary or secondary healthcare level approved for such training in Ogun State. The study sites were chosen because it is believed that any intervention at the level will influence prescribing habits at the other healthcare levels since the HCPs are trained in the facilities.

### Study population, inclusion and exclusion criteria

This study included consecutively selected physicians, pharmacists and nurses at the study settings. Healthcare professionals (Physicians, Pharmacists and Nurses) who attended to older patients with comorbidities were eligible to participate in the study. Neither cluster nor stratified sampling was adopted in the study Eligible participants who were serving at the administrative units, or on rotation in the paediatrics department and those unavailable at the time of the study were excluded from this study.

### Sample size estimation

The sample size was determined using a formula that had been previously described [20]. Based on data obtained from the Ogun State Ministry of Health, there were 4502 HCPs in various hospitals at the time of the study. Using the total population of 4502 at a 95% confidence level, a 5% margin of error, and a 50% response distribution, a minimum sample size of 367 was estimated to be representative of the study population. However, a 20% attrition rate was added giving a maximum sample size of 441.

### Questionnaire design

Questionnaires were developed after the review of some previous studies [15, 21, 22]. Some questions that were considered relevant to practice in developing countries such as Nigeria were prioritised. These include questions on prescribing culture, patients’ factors in deprescribing, interprofessional collaboration, patients’ involvement in decision-making and financial incentives and perversion. The questions were adapted and modified for better comprehension. The self-administered questionnaire was structured into five sections, aimed at covering the scope of the study. Section A consists of five sociodemographic questions including age, gender and profession. Section B consists of five general questions on a Likert scale “Never” to “very frequently” that evaluate the experience of HCPs with deprescribing in older adults.

In section C which consists of 11 questions, the participants were asked to rate the importance of certain considerations for deprescribing in older adults using a 5-point Likert scale “Not important” to “Very important”. Section D consists of nine dichotomous questions that evaluate the participants’ opinions on enablers of deprescribing in older patients. Section E consists of 13 questions on a Likert scale from “strongly agree” to “strongly disagree”, which assessed barriers to deprescribing among the participants (Supplementary 1). The content validity of the questionnaire was determined by two clinical pharmacists with approximately 20 years of experience. The questionnaire was pretested among 20 HCPs in two hospitals in a neighbouring Oyo State. The feedback from pre-test was used to rephrase some questions that appeared to be ambiguous. The Cronbach’s alpha value for the final questionnaire was 0.86.

### Data collection

After appropriate approval was given, copies of the questionnaire were administered to the participants in their various workplaces during their leisure or break-time between the 3rd of April 2022 and the 15th January 2023. The completed copies of the questionnaire were immediately collected from the participants but for those who requested more time, the questionnaire was collected within 48 h.

### Data management and statistical analysis

The data were collected and entered into Microsoft Excel® 2016 (Microsoft, Corp.) and manually cleaned. The data were double-checked for entry error and data completeness, and thereafter, coded. The cleaned data were analyzed via Statistical Package for Social Science (SPSS) version 25 (IBM, Corp.). The 5-point Likert scales were reduced to 3-points during analysis and result presentation for Section C and E. The data were summarised using descriptive statistics including frequency and percentage. The Kruskal–Wallis test, as appropriate, was used to determine differences in perceptions among groups on a Likert scale. A p-value less than 0.05 was considered to be significant.

### Ethical considerations

Ethics approval was obtained from the Ethics Committee of Federal Medical Centre and Olabisi Onabanjo University Ethical Committees, with approval numbers FMCA/470/HREC/01/2023/04 and OOUTH/HREC/453/2021 AP respectively. Gatekeeper permission was obtained from the heads of departments of the study sites. Participants were provided with detailed information about the study, and were assured of confidentiality, anonymity of data to be obtained, and the right to refuse or withdraw from the study. Their written consent was obtained. The study was conducted in line with Helsinki’s declaration.

## Results

### Sociodemographics of the participants

Table 1 presents the sociodemographics of the participants. Of the 500 copies of the questionnaire distributed, only 453 (90.6%) were valid for analysis. The remaining 47 (9.4%) were either not properly filled or contained missing information essential for the analysis. Many of the participants 204 (45.0%) were within the age group of 20–30 years and 266 (55.7%) were female.

**Table 1:** The participants’ sociodemographic characteristics (*n* = 453)

Variable	Frequency	Percentage (%)
Age		
21-30 years	204	45.0
31-40 years	167	36.9
41-50 years	48	10.6
51–60 years	33	7.3
> 60 years	1	0.2
Gender		
Male	187	41.3
Female	266	58.7
Profession		
Medical doctor	193	42.6
Pharmacist	123	27.2
Nurse	137	30.2
Highest educational qualification		
MBBS	152	33.6
B. Pharm	112	24.7
Pharm D	3	0.7
MSc	24	5.3
MD	16	3.5
BNSc	41	9.1
RN	85	18.8
Fellowship	20	4.4
Length of practice		
1-10 years	337	74.4
11-20 years	79	17.4
21-30 years	29	6.4
Above 30 years	8	1.8

*BPharm* bachelor of pharmacy, *MSc* Master of science, *PharmD* Doctor of pharmacy, *PhD* doctor of philosophy, *MBBS* Bachelor of medicine and Bachelor of surgery, *MD* Doctor of medicine, *RN* Registered nurse, *BNSc* Bachelor of nursing science

### The participants’ experience of deprescribing

Table 2 shows the participants’ experience of deprescribing in practice. Many of the participants (193; 42.6%) frequently encounter older adults with comorbidity and polypharmacy and 173 (38.2%) claimed that older adults occasionally request deprescribing of their medications.

**Table 2:** Experience of healthcare professionals about deprescribing in older adults (*n* = 453)

Variable	Never n(%)	Rarely n(%)	Occasionally n(%)	Frequently n(%)	Very frequently n(%)
How often do you see patients who fulfil the following criteria? Age > 65 years, Medication > 5 Chronic diseases > 2					
	3 (0.7)	38 (8.4)	155 (34.2)	193 (42.6)	64 (14.1)
How often are you faced with the challenge of deprescribing in your daily practice with this group?					
	23 (5.1)	107 (23.6)	196 (43.3)	102 (22.5)	25 (5.5)
How often do you de-prescribe medication in the above group of patients?					
	38 (8.4)	118 (26.0)	193 (42.6)	89 (19.6)	15 (3.3)
How often do your patients in this category request for deprescribing of their medications?					
	34 (7.5)	150 (33.1)	173 (38.2)	83 (18.3)	13 (2.9)
How often do pharmacists in your hospital recommend deprescribing medications to you?					
	81 (17.9)	182 (40.2)	134 (39.6)	47 (10.4)	9 (2.0)

### The participants’ considerations for deprescribing in older adults

Table 3 presents the participants’ considerations for deprescribing medications in older adults. The majority (417; 92.1%) expressed the opinion that the patient’s quality of life was very important when deprescribing medications. The overwhelming majority (411; 90.7%) considered patients’ adherence and ability to manage medications very important before deprescribing.

**Table 3:** Healthcare professionals’ considerations for deprescribing in older adults (*N* = 453)

Variable	Not important n(%)	Neutral n (%)	Very important n (%)
Consideration for the medication benefits in a particular patient	33 (7.3)	22 (4.9)	398 (87.9)
The potential risk of medication	19 (4.2)	15 (3.3)	419 (92.5)
Patient’s quality of life	21 (4.6)	15 (3.3)	417 (92.1)
The life expectancy of the patient	33 (7.3)	51 (11.3)	369 (81.5)
The duration of time the patient has been on the medicine	46 (10.2)	39 (8.6)	368 (81.2)
Patient’s preference for to-be de-prescribed medication	101 (22.3)	82 (18.1)	270 (59.6)
Drug interaction	14 (3.1)	28 (6.2)	411 (90.7)
Age of the patient	21 (4.6)	20 (4.4)	412 (90.9)
Intercollaboration with other healthcare practitioners	37 (8.2)	46 (10.2)	370 (81.7)
Patient cognitive impairment	26 (5.7)	54 (11.9)	373 (82.3)
Patient’s adherence and ability to manage medications	13 (2.9)	29 (6.4)	411 (90.7)

### Enablers of deprescribing among HCPs

Table 4 shows the perceptions of healthcare professionals about enablers of deprescribing in older adults. The majority (423; 93.4%) opined that having a care goal known to members of the HCPs team is an enabler for deprescribing while 276 (60.9%) did not consider a reduction in overall health care cost as an enabler for deprescribing among the HCPs.

**Table 4:** Healthcare professionals’ perception of enablers of deprescribing in older patients (*n* = 453)

Variables	Yes n (%)	No n (%)	I am not sure n (%)
Inter-professional medication review is an enabler for deprescribing	444 (98.0)	3 (0.7)	6 (1.3)
Reduction in overall healthcare cost is an enabler for deprescribing	276 (60.9)	122 (26.9)	55 (12.1)
Prescription screening is an enabling factor for deprescribing	396 (87.4)	24 (5.3)	33 (7.3)
Having a care goal known to members of the HCPs team facilitates deprescribing	423 (93.4)	13 (2.9)	17 (3.7)
Effective communication between HCPs is necessary to facilitate deprescribing in older adults	445 (98.2)	6 (1.3)	2 (0.4)
Shared decision-making between patients and HCPs facilitates deprescribing	437 (96.5)	3 (0.7)	13 (2.9)
The availability of simple deprescribing guidelines can facilitate deprescribing	405 (89.4)	14 (3.1)	34 (7.5)
Inclusion of Deprescribing in the curriculum of healthcare practitioners	339 (74.8)	38 (8.4)	76 (16.8)
Point of care tool such as Beers criteria facilitates deprescribing	177 (39.1)	21 (4.6)	255 (56.3)

### Barriers to deprescribing among HCPs

Table 5 presents the participants’ perceptions of barriers to deprescribing. Many of the participants (215; 47.5%) agreed or strongly agreed that the potential negative effect of deprescribing is a barrier to the practice while 308 (68.0%) disagreed or strongly disagreed that lack of incentives and remuneration for healthcare workers that de-prescribe is a barrier to deprescribing.

**Table 5:** Healthcare professionals’ perceptions of barriers to deprescribing in elderly patients (*N* = 453)

Variable	Strongly agree/agree n(%)	Neutral n(%)	Disagree /disagree n (%)
The non-emphasis on deprescribing in the healthcare training curriculum is a barrier to deprescribing	328 (72.4)	48 (10.6)	77 (17.0)
Lack of validated algorithms for deprescribing	301 (66.4)	92 (20.3)	60 (13.2)
The potential negative effect of deprescribing	215 (47.5)	136 (30.0)	102 (22.5)
Deprescribing a medication prescribed by other prescribers is problematic	246 (54.3)	2 (0.4)	205 (45.3)
Managing the expectations of the patients/relatives	248 (54.7)	5 (1.1)	200 (44.2)
Multiple guidelines for managing comorbidity in older adults	262 (57.8)	5 (1.1)	186 (41.1)
Lack of incentives and remuneration for healthcare workers that de-prescribe	135 (29.8)	10 (2.2)	308 (68.0)
Ethical and legal issues involved in deprescribing	262 (57.8)	106 (23.4)	85 (18.8)
Insufficient evidence to support the benefit of deprescribing	185 (40.8)	113 (24.9)	155 (34.2)
Restricted authority to de-prescribe medication by only the specialists	268 (59.2)	75 (16.6)	110 (24.3)
The willingness of patients to accept deprescribing	296 (65.3)	77 (17.0)	80 (17.7)
Pressure from pharmaceutical companies	197 (43.5)	87 (21.4)	159 (35.1)
Patients not taking an active role in decision-making concerning their medication management	313 (69.1)	68 (15.0)	72 (15.9)

### Association between professional groups and participants’ opinions on important considerations for deprescribing among older adults

Table 6 presents the association between respondents’ opinions on deprescribing and their profession. There was a significant difference in the opinions of healthcare professionals across professional groups regarding patient cognitive impairment as a consideration in deprescribing (*p* = 0.032). The respondents’ profession was not significantly associated with their responses concerning the importance of intercollaboration among healthcare practitioners as a consideration in deprescribing (*p* = 0.134).

**Table 6:** Association between respondents’ profession and considerations in deprescribing

Variables	Not important n (%)	Neutral n (%)	Very important n (%)	Mean Rank	Median IQR	KW-*p*value
Consideration for the medication benefits in a particular patient						
Physician	7(1.5)	13(2.9)	173(38.2)	231.74		
Pharmacist	10 (2.2)	6 (1.3)	107 (23.6)	224.95	3 (3–3)	0.481
Nurse	16 (3.5)	3 (0.7)	118 (26.0)	222.16		
The potential risk of medication is considered in deprescribing						
Physician	8(1.8)	4(0.9)	181(40)	229.80		
Pharmacist	7 (1.5)	5 (1.1)	111 (24.5)	221.86	3(3–3)	0.509
Nurse	4 (0.9)	6 (1.3)	127 (28.0)	227.66		
Patient’s quality of life is a factor						
Physician	8(1.8)	2(0.4)	183(40.4)	233.06		
Pharmacist	8 (1.8)	4 (0.9)	111 (24.5)	222.76	3 (3–3)	0.194
Nurse	5 (1.1)	9 (2.0)	123 (27.3)	222.27		
The life expectancy of the patient						
Physician	15(3.3)	27(6.0)	151(33.3)	220.04		
Pharmacist	7 (1.5)	15 (3.3)	101 (22.3)	229.65	3 (3–3)	0.306
Nurse	11 (2.4)	9 (2.0)	117 (25.8)	234.97		
The duration of time the patient has been on the medicine						
Physician	20(4.4)	20(4.4)	153(33.8)	222.92		
Pharmacist	12 (2.6)	12 (2.6)	99 (21.9)	225.65	3(2–3)	0.529
Nurse	14 (3.1)	7 (1.5)	116 (25.6)	233.96		
Patient’s preference for about to be deprescribed						
Physician	49(10.8)	39(8.6)	105(23.2)	215.02		
Pharmacist	24 (5.3)	20 (4.4)	79 (17.4)	237.69	3 (3–3)	0.157
Nurse	28 (6.2)	23 (5.1)	86 (19.0)	234.28		
Drug interaction						
Physician	3(0.7)	13(2.9)	177(39.1)	229.48		
Pharmacist	2 (0.4)	7 (1.5)	114 (25.2)	231.60	3 (3–3)	0.258
Nurse	9 (2.0)	8 (1.8)	120 (26.5)	219.38		
Age of the patient						
Physician	12(2.6)	9(2.0)	172(38.0)	222.72		
Pharmacist	2 (0.4)	6 (1.3)	115 (25.4)	233.12	3 (3–3)	0.382
Nurse	7 (2.0)	5 (1.1)	125 (27.6)	227.53		
Intercollaboration among healthcare practitioners						
Physician	15(3.3)	23(5.1)	155(34.2)	224.32		
Pharmacist	4 (0.9)	12 (2.6)	197 (23.6)	240.09	3 (3–3)	0.134
Nurse	18 (4.0)	11 (2.4)	104 (23.8)	219.02		
Patient cognitive impairment						
Physician	6(1.3)	25(5.5)	162(35.8)	231.46		
Pharmacist	5 (1.1)	11 (2.4)	107 (23.6)	237.60	3 (3–3)	0.032
Nurse	15 (3.3)	18 (4.0)	108 (23.8)	211.19		
Patient’s adherence and ability to manage medications						
Physician	3(0.7)	11(2.4)	179(39.5)	231.72		
Pharmacist	1 (0.2)	8 (1.8)	114 (25.2)	231.73	3(1–3)	0.068
Nurse	9 (2.0)	10 (2.2)	118 (26.0)	216.11		

*P* < 0.05 is considered significant, *IQR* interquartile range, *KW* Kruskal–Wallis

### Association of participants’ perceptions of barriers to deprescribing with their professions

Table 7 presents the association between the profession and participants’ responses to barriers to deprescribing in older patients. There was a significant difference in the opinions of the participants across the professional groups concerning the assertion that “pressure from pharmaceutical companies is a barrier to deprescribing” (*p* = 0.037). The professionals’ opinions were not significantly different with regard to the willingness of patients to accept deprescribing as a barrier to the practice (*p* = 0.344).

**Table 7:** Association between participants’ response and profession on barriers to deprescribing (*n* = 453)

Variable	SD/D n (%)	Neutral n (%)	SA/A n (%)	Mean Rank	Median IQR	KW-*P*value
The non-emphasis on deprescribing in the healthcare training curriculum is a barrier to deprescribing						
Physician	39 (8.6)	14 (3.1)	40 (30.9)	228.76		
Pharmacist	12 (2.6)	11 (2.4)	100 (22.1)	205.75	1(2–1)	0.012
Nurse	26 (5.7)	23 (5.1)	88 (19.4)	243.60		
Lack of validated tool for deprescribing						
Physician	23 (5.1)	47 (10.4)	123 (27.2)	231.33		
Pharmacy	6 (1.3)	26 (5.7)	91 (20.1)	205.83	1 (1–2)	0.033
Nurse	31 (5.1)	19 (4.2)	87 (19.2)	239.91		
The potential negative effect of deprescribing						
Physician	46 (10.2)	65 (14.3)	82 (18.1)	237.30		
Pharmacy	27 (6.0)	42 (9.3)	54 (11.9)	232.57	2 (1–2)	0.074
Nurse	29 (6.4)	29 (6.4)	79 (17.4)	207.49		
Deprescribing a medication prescribed by other prescribers is problematic						
Physician	84 (18.5)	1 (0.2)	108 (23.8)	223.16		
Pharmacist	52 (11.5)	1 (0.2)	70 (15.5)	220.69	1 (1–3)	0.383
Nurse	69 (15.2)	0 (0.0)	68 (15.0)	238.08		
Managing the expectations of the patients/relatives						
Physician	83 (18.3)	1 (0.2)	109 (24.1)	223.64		
Pharmacist	51 (11.3)	4 (0.9)	68 (15.0)	223.57	1 (1–3)	0.627
Nurse	66 (14.6)	0 (0.0)	71 (15.7)	234.82		
Multiple guidelines for managing comorbidity in the elderly						
Physician	82 (18.1)	3 (0.7)	108 (23.8)	230.87		
Pharmacist	51 (11.3)	2 (0.4)	70 (15.5)	228.62	1 (1–3)	0.680
Nurse	53 (11.7)	0 (0.0)	84 (18.5)	220.09		
Lack of incentives and remuneration for healthcare workers that de-prescribe						
Physician	139 (30.7)	3 (0.7)	51 (11.3)	235.85		
Pharmacist	76 (16.8)	6 (1.3)	41 (9.1)	214.58	3 (1–3)	0.219
Nurse	93 (20.5)	1 (0.2)	43 (9.5)	225.68		
Ethical and legal issues involved in deprescribing						
Physician	38 (8.4)	63 (13.9)	92 (20.3)	246.59		
Pharmacist	23 (5.1)	28 (6.2)	72 (15.9)	225.65	1 (1–3)	0.002
Nurse	24 (5.3)	15 (3.3)	98 (21.6)	200.61		
There is no sufficient evidence to support the benefit of deprescribing						
Physician	76 (16.8)	51 (11.3)	66 (14.6)	243.81		
Pharmacist	36 (7.9)	42 (9.3)	45 (9.9)	226.71	2 (1–3)	0.013
Nurse	43 (9.5)	20 (4.4)	74 (16.3)	203.58		
The authority to deprescribe unnecessary medication resides with the specialists						
Physician	43 (9.5)	38 (8.4)	112 (24.7)	227.09		
Pharmacist	25 (5.5)	20 (4.4)	78 (17.2)	216.04	1(1–2)	0.352
Nurse	42 (9.3)	17 (3.8)	78 (17.2)	236.721		
The willingness of patients to accept deprescribing						
Physician	34 (7.5)	42 (9.3)	117 (25.8)	235.77		
Pharmacist	19 (4.2)	20 (4.4)	84 (18.5)	219.76	1 (1–2)	0.344
Nurse	27 (6.0)	15 (3.3)	95 (21.0)	221.15		
Pressure from pharmaceutical companies						
Physician	79 (17.4)	42 (9.3)	72 (15.9)	243.55		
Pharmacist	33 (7.3)	31 (6.8)	59 (13.0)	209.83	2 (1–3)	0.037
Nurse	47 (10.4)	24 (5.3)	66 (14.6)	219.09		
Patients not taking an active role in decision-making concerning their medication management						
Physician	28 (6.2)	34 (7.5)	131 (28.9)	228.35		
Pharmacist	19 (4.2)	19 (4.2)	85 (18.8)	226.67	1 (1–2)	0.969
Nurse	25 (5.5)	15 (3.3)	97 (21.4)	225.39		

*P* < 0.05 is considered significant, *IQR* interquartile range, *KW* Kruskal–Wallis

## Discussion

This study aimed to explore the considerations, barriers and enablers of deprescribing in older patients among HCPs in Nigeria. The study found that participants had various considerations for deprescribing medication in older adults including patients’ quality of life. The existence of a care goal known to the HCPs team and shared decision-making between patients and HCPs are enablers of deprescribing while the lack of incentives and remuneration for HCPs that deprescribe was not considered a barrier to deprescribing.

In this study, nearly two out of five participants claimed that older multimorbid patients with polypharmacy occasionally request deprescribing. This is an indication that patients may not be averse to deprescribing especially if initiated by a trusted practitioner. The description of deprescribing as “swimming against the tide” of patient expectation may therefore not be true [23]. The finding of this study is in tandem with the views expressed by older patients in similar studies in resource-limited settings [14, 18, 24]. The present study revealed that pharmacists never or rarely recommend deprescribing to physicians or nurses judging by the opinions of more than half of the participants. This finding is contrary to the expectation of other healthcare providers who consider pharmacists’ role as very key to the success of any deprescribing intervention [21]. Pharmacists through medication review can identify medications that require deprescribing and assist in addressing important patient barriers including resistance to deprescribing and improving knowledge of medications [25]. However, the observation of this study may be due to fear of conflict between pharmacists and other healthcare providers or uncertainty about their roles in deprescribing [13, 22, 26].

The majority of the HCPs in this study considered patient’s quality of life, life expectancy, potential risk of medication and patients’ adherence and ability to manage medications as very important factors that could determine their deprescribing behaviour. Evidence suggests that consideration of the aforementioned factors is germane to successful deprescribing interventions in older patients [4, 25]. The findings of this study probably suggest that HCPs in Nigeria are aware of the conditions for deprescribing but may be limited in their practice by factors other than knowledge.

Many barriers to deprescribing including challenges in communication among HCPs and lack of access to patients’ records have been reported in the literature [3, 21, 22]. In this study, the majority of the participants believed that having a care goal known to every HCP involved in managing patients creates an enabling condition for deprescribing. This finding is significant and underscores the importance of clear communication between healthcare providers, interprofessional collaboration and unhindered access to patient records by every member of the team. This study’s participants agreed that the active involvement of patients in decision-making is an enabler for deprescribing. This finding is consistent with reports in many well-resourced countries [13, 22, 25]. It is worth noting that in this study many HCPs did not believe that a reduction in the health care costs is an enabler of deprescribing. This contradicts the observation of a study in Ireland [27]. The reasons for the findings of the present study may need to be further investigated.

The multiple guidelines for managing multimorbidity in older patients and managing the expectations of patients and relatives and specialists’ authority on prescription were considered to be barriers to deprescribing by many of the HCPs in this study. These findings are consistent with reports of similar studies [13, 22, 23]. In this study, there was a difference in opinions among the participants regarding the assertion that pressure from pharmaceutical companies is a barrier to deprescribing. The influence of pharmaceutical companies appears to be a malady and a hindrance to effective prescribing especially in resource-limited settings [28, 29]. Many of the participants agreed that ethical and legal issues were barriers to deprescribing similar to a report among physicians in the United States of America [30].

The majority of the participants in this study strongly disagreed or disagreed that the lack of incentives and remuneration for HCPs who de-prescribe is a barrier to deprescribing. This finding contradicts a similar study in New Zealand that reported adequate reimbursement as an enabler for deprescribing among general practitioners [21]. The present finding corroborates the assertion that findings about barriers and enablers in developed countries may not be extrapolated to resource-limited settings, hence the need for studies in such countries [17]. In Nigeria, the remuneration of healthcare professionals is fixed and healthcare costs are largely out-of-pocket. There is no additional incentive for proper prescribing habits as obtainable in some developed countries that operate national health insurance schemes.

### Strengths and limitations of the study

This study highlights the considerations and priorities that guide deprescribing among HCPs in a resource-limited country such as Nigeria. The inclusion of HCPs from different professions in this study provided an integrated perspective of the barriers and enablers of deprescribing in a complex healthcare system. This may allow more robust strategies and policies to be developed to enhance deprescribing practices among HCPs.

However, the study was carried out in two tertiary healthcare facilities in Ogun state alone, the respondent sample may not be representative of all HCPs or regions thus, limiting the generalizability of the results. The perceptions of HCPs in other layers of healthcare delivery may be different. A quantitative survey method was used which may not have provided an in-depth exploration of the reasoning behind the different responses. The sampling technique adopted in this study could have skewed the demographic characteristics and thus limited the generalisability of the results.

### Implications for research and practice

This study asserts the importance of a multidisciplinary approach involving pharmacists and nurses and adequate sharing of patients’ information and care goals in the care of older people among HCPs. The Nigerian standard treatment guidelines need to mention and emphasise deprescribing process and where it is required. Healthcare policy-makers can leverage the findings of this study and similar ones to give more clinical roles to pharmacists including medication review since this appears to be the expectation of other healthcare professionals in Nigeria. Pharmacists-led medication review will free up more time for physicians to attend to other patients, and this will benefit the healthcare system in a country such as Nigeria where physicians are not adequate in number.

The HCP’s skills on how to manage the expectations of patients through communication is a sine-qua non for an effective deprescribing process. This may require additional training with practical guidance for some HCPs. The training of healthcare professional students should incorporate hands-on training on effective communication between older patients/caregivers and HCPs. The de-prescribing process and guidelines should be taught in the same manner as medication prescribing in the HCPs’ curriculum.

The existing healthcare laws in the country may need to be reviewed to give more clear roles to other healthcare workers, especially in the care of older persons, and to protect physicians willing to de-prescribe medications. There may be the need to review medicine laws guiding the detailing of pharmaceuticals to physicians in the hospitals. Although this study did not consider remuneration as a barrier to deprescribing in Nigeria, research on the cost-effectiveness of the process and its economic advantage to the healthcare system in Nigeria which is currently lacking, may provide an impetus for the government to consider remunerating HCPs for deprescribing, thereby encouraging the intervention.

### Conclusions

The participants in this study had various considerations for deprescribing medication in older adults including patients’ quality of life and patients’ adherence and ability to manage medications. The enablers for deprescribing in this study include having a care goal known to every HCP involved in managing a patient and sharing decision-making between patients and HCPs. The lack of incentives and remuneration for HCPs that deprescribe may not necessarily be a barrier to deprescribing in Nigeria. There is a need for regulations and policies to support the identified enablers among HCPs and reduce the barriers to effective deprescribing processes. A wider qualitative study with national geographic spread focussing on the possibility of processes and policy changes to address barriers and enablers of deprescribing in Nigeria is advocated.

### Supplementary Information

Supplementary Material 1.

## Data Availability

All data generated or analysed during this study are included in this published article [and its supplementary information files].

## Abbreviations

HCPs: Healthcare Professionals
PIMs: Potential Inappropriate Medications
OOUTH: Olabisi Onabanjo University Teaching Hospital
FMC: Federal Medical Centre
HREC: Health Research Ethics Committee
