# Supplementary material for: Considerations, barriers and enablers of deprescribing among healthcare professionals in Ogun State, Southwest, Nigeria: a cross-sectional survey
Source: BMC Health Serv Res. 2024 May 24;24:661. doi: 10.1186/s12913-024-11101-0 (PMC11127322; doi:10.1186/s12913-024-11101-0)
Supplement: Supplementary file 1 — Supplementary Material 1. [file 12913_2024_11101_MOESM1_ESM.docx]

Dear Sir/ma,

This survey is to elicit your opinion about deprescribing medications in older patients. They are primarily for research purpose. Your responses will be treated with utmost confidentiality and anonymity. The survey will take about 10 minutes and your sincere answer to each of the question will be highly appreciated. Thank you

Do you consent to participate in this study? Yes [ ] No [ ]

Instruction: Kindly choose the option that best applies to you in the appropriate sections

**Section A: Participants’ socio-demographic-**

Age: 20-30years [ ] 31-40 years [ ] 41-50 years [ ] 51-60 years [ ] above 60 years [ ]

Gender: Male [ ] Female [ ]

Profession: Medical doctor [ ] Pharmacist [ ] Nurse [ ]

Highest educational qualification: MBBS [ ] B Pharm. [ ] RN [ ] MSc [ ] MD [ ] Fellowship [ ] PhD [ ]

Length of practice: 1-10 years [ ] 11-20 years [ ] 21-30 years [ ] above 30 years [ ]

**Section B: The healthcare professionals’ experience of deprescribing in elderly patients**

| S/N | Question | Never | Rarely | Occasionally | frequently | Very frequently |
| --- | --- | --- | --- | --- | --- | --- |
| 1 | How often do you see patients who fulfil the following criteria?   - Age ≥65 years - Medication ≥5 - Chronic diseases >2 |  |  |  |  |  |
| 2 | How often are you faced with the challenge of deprescribing in your daily practice with this group of patients? |  |  |  |  |  |
| 3 | How often do you deprescribe medication in the above group of patients? |  |  |  |  |  |
| 4 | How often do your patients in this category request deprescribing of their medications? |  |  |  |  |  |
| 5 | How often do pharmacists in your hospital recommend deprescribing of medications to you? |  |  |  |  |  |

**Section C: Healthcare professionals’ opinions on deprescribing in elderly patients**

Kindly rate the importance of the following factors to deprescribing in older persons

| **S/N** | **Question** | **Not important** | **Fairly important** | **Neutral** | **Important** | **Very important** |
| --- | --- | --- | --- | --- | --- | --- |
| 11 | Consideration for the medication benefit in a particular patient |  |  |  |  |  |
| 12 | The potential risk of medication is considered in deprescribing |  |  |  |  |  |
| 13 | Patients’ quality of life is a factor |  |  |  |  |  |
| 14 | The life expectancy of the patient |  |  |  |  |  |
| 15 | The duration of time the patient has been on the medication |  |  |  |  |  |
| 16 | Patient’s preference for about to be deprescribed medication |  |  |  |  |  |
| 17 | Drug interaction |  |  |  |  |  |
| 18 | Age of the patient |  |  |  |  |  |
| 19 | Inter collaboration with other healthcare practitioners |  |  |  |  |  |
| 20 | Patient cognitive impairment |  |  |  |  |  |
| 21 | Patients’ adherence and ability to manage medications |  |  |  |  |  |

**Section D: The healthcare professionals’ opinions on enablers for deprescribing in elderly patients**

| S/N | Question | Yes | No | I am not sure |
| --- | --- | --- | --- | --- |
| 22 | Inter-professional medication review is a facilitator for deprescribing |  |  |  |
| 23 | Reduction in overall healthcare cost is a facilitator for deprescribing |  |  |  |
| 24 | Prescription screening is an enabling factor for deprescribing |  |  |  |
| 25 | Having a care goal known to members of the HCPs team facilitates deprescribing |  |  |  |
| 26 | Effective communication between the healthcare professionals is necessary to facilitate deprescribing in older patients |  |  |  |
| 27 | Share-decision making between patients and HCPs facilitates deprescribing |  |  |  |
| 28 | The availability of simple deprescribing guidelines can facilitate deprescribing |  |  |  |
| 29 | Inclusion of deprescribing in the curriculum of healthcare practitioners |  |  |  |
| 30 | Point of care tool such as Beers criteria facilitates deprescribing |  |  |  |

**Section E: Healthcare professionals’ perceptions of barriers to deprescribing in elderly patients**

**SA-strongly agree, A-agree, N-neutral, D-disagree, SD-strongly disagree**

| **S/N** | **Question** | **SD** | **D** | **N** | **A** | **SA** |
| --- | --- | --- | --- | --- | --- | --- |
| 31 | The non-emphasis on deprescribing in the healthcare training curriculum is a barrier to deprescribing |  |  |  |  |  |
| 32 | Lack of validated tool for deprescribing |  |  |  |  |  |
| 33 | The potential negative effect of deprescribing |  |  |  |  |  |
| 34 | Deprescribing a medication prescribed by other prescribers is problematic |  |  |  |  |  |
| 35 | Managing the expectation of the patients/ relatives |  |  |  |  |  |
| 36 | Multiple guidelines for managing comorbidity in the elderly |  |  |  |  |  |
| 37 | Lack of incentives and remuneration for healthcare workers that deprescribe |  |  |  |  |  |
| 38 | Ethical and legal issues involved in deprescribing |  |  |  |  |  |
| 39 | There is no sufficient evidence to support the benefit of deprescribing |  |  |  |  |  |
| 40 | Restricted authority to de-prescribe medication by only the specialists is a barrier |  |  |  |  |  |
| 41 | The willingness of patients to accept deprescribing |  |  |  |  |  |
| 42 | Pressure from pharmaceutical companies |  |  |  |  |  |
| 43 | Patients not taking an active role in decision making concerning their medication management |  |  |  |  |  |

Thank you for your response
